# Clinical thresholds for diagnosing iron deficiency: comparison of functional assessment of serum ferritin to population based centiles

**DOI:** 10.1038/s41598-020-75435-5

**Published:** 2020-10-26

**Authors:** Gorkem Sezgin, Paul Monagle, Tze Ping Loh, Vera Ignjatovic, Monsurul Hoq, Christopher Pearce, Adam McLeod, Johanna Westbrook, Ling Li, Andrew Georgiou

**Affiliations:** 1 Centre for Health Systems and Safety Research, Australian Institute of Health Innovation, Faculty of Medicine, Health and Human Sciences, Macquarie University, Level 6, 75 Talavera Road, Ryde, NSW 2109 Australia; 2 Department of Pediatrics, The University of Melbourne, Parkville, VIC Australia; 3 Hematology Research, Murdoch Children’s Research Institute, Parkville, VIC Australia; 4 Department of Hematology, Royal Children’s Hospital Melbourne, Parkville, VIC Australia; 5 Department of Laboratory Medicine, National University Hospital, Kent Ridge, Singapore; 6 Clinical Epidemiology and Biostatistics Unit, Murdoch Children’s Research Institute, Parkville, VIC Australia; 7 Outcome Health, East Burwood, VIC Australia

**Keywords:** Diagnostic markers, Paediatric research, Statistical methods, Iron

## Abstract

Low serum ferritin is diagnostic of iron deficiency, yet its published lower cut-off values are highly variable, particularly for pediatric populations. Lower cut-off values are commonly reported as 2.5th percentiles, and is based on the variation of ferritin values in the population. Our objective was to determine whether a functional approach based on iron deficient erythropoiesis could provide a better alternative. Utilizing 64,443 ferritin test results from pediatric electronic health records, we conducted various statistical techniques to derive 2.5th percentiles, and also derived functional reference limits through the association between ferritin and erythrocyte parameters: hemoglobin, mean corpuscular volume, mean cell hemoglobin concentration, and red cell distribution width. We find that lower limits of reference intervals derived as centiles are too low for clinical interpretation. Functional limits indicate iron deficiency anemia starts to occur when ferritin levels reach 10 µg/L, and are largely similar between genders and age groups. In comparison, centiles (2.5%) presented with lower limits overall, with varying levels depending on age and gender. Functionally-derived limits better reflects the underlying physiology of a patient, and may provide a basis for deriving a threshold related to treatment of iron deficiency and any other biomarker with functional outcomes.

## Introduction

Iron deficiency is the major cause of anemia that affects up to 32.9% of the global population, with the highest burden seen in young children^1^. Iron requirements are increased during rapid growth of early childhood, and during adolescence for females, making these population groups particularly vulnerable^2^. Untreated iron deficient erythropoiesis can lead to anaemia^3^, which is associated with reduced cognitive development among children^4^.

Although a variety of laboratory assessments for iron status is available, serum ferritin provides the highest diagnostic accuracy in relation to the gold standard, bone marrow aspirate^5^. Low serum ferritin is pathognomonic of uncomplicated iron deficiency^6^. Serum ferritin tests are commonly accompanied by erythrocyte parameters: hemoglobin, mean corpuscular volume (MCV), mean corpuscular hemoglobin (MCH), mean cell hemoglobin concentration (MCHC), and red cell distribution width (RDW), when investigating iron deficiency anemia. Iron deficiency anemia is characterized with a reduction in all erythrocyte parameters except RDW^6^.

Thresholds for defining iron deficiency based on serum ferritin are highly variable, particularly for children^7–9^. Ideally, reference intervals are determined from healthy populations and stratified according to age, gender, and other physiological states when they show distinct subpopulation distributions^10^. Establishing reference intervals for children is a difficult task, with significant logistic and resource challenges in collecting samples from a large population of healthy children. Nevertheless, a number of international initiatives have established robust and nationally harmonized reference intervals; a prominent example being the *Canadian laboratory initiative on pediatric reference intervals* (CALIPER) study^11^. Most reference interval harmonization initiatives follow the *direct approach* recommended by Clinical and Laboratory Standards Institute (CLSI), with 2.5th and 97.5th percentiles calculated as reference intervals from a ‘healthy’ population^12,13^.

In recent years the *indirect approach* of deriving reference intervals has gained interest, where secondary data sources such as hospital pathology records^14^ and pathology laboratory databases^15^ are explored as alternative populations^13^. Through use of stringent inclusion criteria to derive a ‘healthy’ population^16^ and/or through novel statistical methods^17^, secondary data sources are proving to be a viable option.

Regardless of the approach, reference intervals dependent on measured values within the population selected. If iron deficiency is highly prevalent, which may be asymptomatic, then both direct and indirect approaches will under-estimate the lower limit of “normal”.

Through a novel approach based on the functional association between serum ferritin and erythrocyte parameters, studies have identified serum ferritin thresholds with clinical relevance to iron deficiency anaemia^18,19^. Our objective was to determine whether a functional approach based on clinically relevant iron deficient erythropoiesis could provide a more clinically useful alternative to direct and indirect approaches.

## Methods

### Data source

In this study, we used de-identified pathology data from 211 general practices collected from the *Population Level Analysis and Reporting* (POLAR) data space, provided by the data custodians, Outcome Health^20^. Outcome Health collects data on behalf of primary health networks (PHNs), which are geographically localized health districts aiming to improve provision of healthcare services. In this study, data from three PHNs from Victoria, Australia: South-Eastern Melbourne, Eastern Melbourne, and Gippsland were used.

Our analyses included all laboratory results of ferritin (µg/L and ng/mL, standardized to µg/L), hemoglobin (g/L), MCV (fL), MCHC (g/L), MCH (pg), and RDW (%) measured between 2008 and 2018 for those aged 2 to 18 years, as identified by test names and *Logical Observation Identifiers Names and Codes* (LOINC)^21^. Biomarkers were assessed for differences in measurement years by investigating variation in medians and inter-quartile ranges. No significant variations were observed.

### Exclusion/inclusion criteria

Ferritin is a positive acute phase reactant, and can increase during acute infection/inflammation, even in iron deficiency. C-reactive protein (CRP), an inflammation marker, is helpful in interpreting ferritin results in such cases, although the criteria used to define iron deficiency during infection/inflammation not well established^22^. Therefore, results associated with increased CRP results above reported reference intervals (as ≥ 10 mg/L^23,24^), were excluded. Furthermore, participants who had a diagnostic record for thalassemia, and participants on iron supplement medication within the last year of the test were excluded. If several tests were recorded for a participant, only the earliest record was included.

### Statistical analyses

Various approaches were used to determine lower reference limit for ferritin.

### Clinical and Laboratory Standards Institute (CLSI) parametric approach

In this analysis, the lower reference limit was derived according to principles outlined in CLSI guidelines, as reported by Solberg^25^. The dataset was stratified by gender, as recorded in patients’ electronic health records, and the lower reference limit was determined for each age between 2 to 18 years (calculated as: year at time of laboratory test – year of birth).

Initially, distribution of ferritin values were plotted as a scatterplot by gender and age. The data was transformed to a Gaussian distribution through Box–Cox^26^ transformation at each age value. A presentation of the overall distribution of serum ferritin values, and an overall Box–Cox transformation reducing the skew of the distribution can be found in supplementary figure S1. Ferritin results ≥ 1000 µg/L were excluded from further analysis. Remaining data were subjected to outlier detection according to Tukey’s method^27^, where values lying outside of ‘outer fences’ were excluded:

$$ below\; 25th\; percentile - (3 \times IQR) $$

or

$$ above\; 75th\; percentile + (3 \times IQR) $$

Tukey’s outlier removal was performed in two replicates for each age and gender category. Distribution of remaining data was evaluated by Q–Q (quantile–quantile) plots. Lastly, parametric fractiles at each year of age by gender, and their respective 90% confidence intervals were estimated using the following formulas^25^, respectively:

$$ lower\; fractile = mean - 1.960 \times SD $$

$$ 90\% CI = fractile \; \pm \;2.81 \times SD\surd N $$

Final values were converted back into their original forms by reversing Box–Cox transformations.

### Clinical and Laboratory Standards Institute (CLSI) non-parametric (rank method) approach

This analysis follows the rank-based procedure outlined by Solberg^25^. We determined rank-based fractiles for the lower-level of the reference interval for ferritin, stratified by gender for ages between 2 and 18 years. Initially, results values were sorted from lowest to highest. The position of the lower fractile in the rank was determined using the following formula:

$$ 0.025 \times (N + 1) $$

Results obtained from this formula determined the position where the lower reference lies in the sorted list of ferritin test results.

### Multivariate fractional polynomial

In this analysis, a non-parametric approach to derive the 2.5th percentile for ferritin was used. For each gender category, bivariate fractional polynomial models based on median for the association between ferritin test results and age were formed, with quantile regression at the 2.5th percentile calculated. Based on these models, the 2.5th percentiles for each year of age between 2 and 18 were estimated using post-estimation calculations, including their respective 95% confidence intervals.

### Correlation between serum ferritin and erythrocyte parameters

By using a similar approach in the study performed by Markus et al*.*^18^, we modelled the correlation between ferritin and erythrocyte parameters: hemoglobin, MCV, MCHC, MCH and RDW. The rationale in this approach is that reduced iron stores, as reflected by serum ferritin concentrations are followed by a reduction in hemoglobin, MCV, MCHC and MCH, and an increase in RDW as it progresses towards anaemia^6^.

The best model to describe the association between ferritin and erythrocyte parameters were explored through scatterplots and median-spline plots. We determined that 2-degree fractional polynomial models would best describe the correlations, and multivariate fractional polynomials were plotted, modelling quantile regression at medians (Figs. 2, 3, 4, 5, 6).

Our models were stratified by age groups and gender. Ages were categorized into three groups: ages 2–4, 5–12, and 13–8 years, similar to those described by Markus el al.^18^. We then estimated the erythrocyte value at each ferritin concentration from 1 to 60 µg/L, including their respective 95% confidence intervals, and plotted values for each age and gender group.

We determined the point where iron deficiency starts to progress to anemia based on the correlation between ferritin and erythrocyte parameters beginning to asymptote. Asymptote occurred when erythrocyte value differences between subsequent correlated ferritin values became less than 1 (to one decimal place). All statistical analyses were conducted using Stata/MP 16.0 (StataCorp., TX, USA).

### Ethics

Ethics to extract and analyze de-identified general practice data for research was obtained by data custodians of the POLAR data space from RACGP National Research and Evaluation Ethics Committee (NREEC 17–008). Our research team obtained ethics to conduct research on these data from Macquarie University Human Research Ethics Committee (5201700872). Informed consent from patients was not required, as approved by both RACGP National Research and Evaluation Ethics Committee and Macquarie University Human Research Ethics Committee.

Our study was conducted and reported according to the Reporting of Studies Conducted Using Observational Routinely Collected Health Data Statement for Pharmacoepidemiology (RECORD-PE) guideline^28^.

## Results

**Figure 1:**
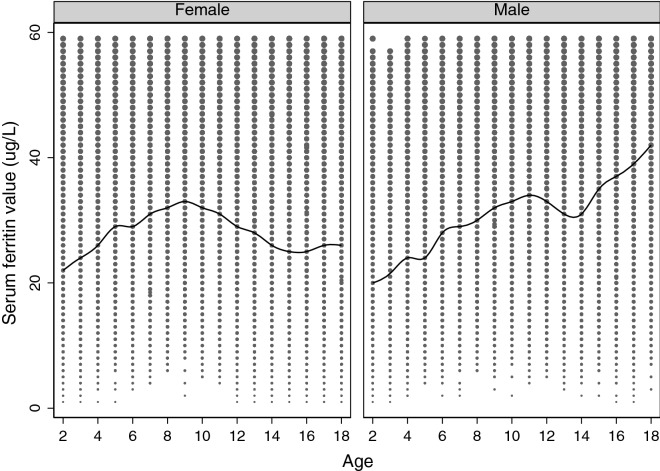
Scatterplot and median spline of the distribution of serum ferritin values by age and gender. Serum ferritin results up to 60 µg/L are shown.

Number of test results included in each analysis is summarized in Table 1. A total of 39,910 ferritin tests were ordered for females, and 24,533 for males. Figure 1 shows the different distributions of serum ferritin test results between females and males for serum ferritin results < 60 µg/L. Parametric and rank-based CLSI results presented similar distributions for both genders until age of 10, after which females had markedly lower values compared to males (Tables 2, 3). Similar results were observed for our fractional polynomial model, with females having lower values beginning at age 7 compared to males. Overall, males had a relatively linear increase in ferritin values with age, whereas for females, an increase was observed until near adolescence, after which a decrease occurred.

**Table 1:** Number of ferritin test results included in each age and gender groups included in the analyses.

Age	Female						Male					
	Frequency	With Hb	With MCV	With MCH	With MCHC	With RDW	Frequency	With Hb	With MCV	With MCH	With MCHC	With RDW
2	599	2064	2061	1898	1682	1634	851	2754	2746	2542	2231	2224
3	731						1010					
4	855						1078					
5	955	9939	9893	9033	8144	8027	1185	9456	9418	8565	7783	7455
6	1038						1083					
7	1198						1221					
8	1223						1256					
9	1299						1201					
10	1383						1290					
11	1499						1345					
12	1772						1308					
13	2602	26,208	26,100	22,947	20,740	19,733	1580	11,204	11,167	9885	8821	8623
14	3323						1725					
15	4358						1863					
16	5189						1988					
17	5950						2292					
18	5936						2257					
Total	39,910	38,211	38,054	33,878	30,566	29,394	24,533	23,414	23,331	20,992	18,835	18,302

Number of ferritin results accompanied by erythrocyte parameters also described.

*Hb* hemoglobin, *MCV* mean corpuscular volume, *MCH* mean corpuscular hemoglobin, *MCHC* mean corpuscular hemoglobin concentration, *RDW* red cell distribution width.

**Table 2:** Lower reference limits (2.5th percentile) for ferritin in µg/L among pediatric patients for females.

Age	CLSI Parametric	CLSI Rank	Fractional polynomial	Correlation between ferritin and erythrocyte parameters					Hoq et al*.*^33^					Adeli et al*.*^11^		
				Hb	MCV	MCH	MCHC	RDW	Roche e602	Abbott	Roche c702	Siemens	Beckman	Abbott	Beckman	Ortho 5600
2	4.3	5	2.5	10	8	5	11	5	12.5	10.1	8.5	9	9	5	10.3	9.51
3	5.0	4	6.3						10.7	8.1	6.8	7.3	7.3			
4	7.2	7	8.0						9.4	6.8	5.7	6.1	6.1			
5	9.1	8	8.8	10	6	4	10	5	8.6	6	4.9	5.4	5.4	14		
6	9.6	9	9.1						8	5.4	4.2	4.8	4.8			
7	10.5	10	9.2						7.6	5	3.8	4.4	4.4			
8	11.8	11	9.0						7.3	4.8	3.4	4.2	4.1			
9	12.7	13	8.7						7.1	4.7	3.1	4	3.9			
10	11.0	10	8.4						7	4.7	2.9	3.9	3.8			
11	10.9	10	8.0						7	4.7	2.8	3.9	3.8			
12	7.8	7	7.5						7	4.8	2.6	3.9	3.8			
13	7.3	7	7.0	11	7	5	9	3	7	5	2.6	3.9	3.8			8.28
14	5.8	5	6.5						7.1	5.2	2.5	4	3.9			
15	5.9	6	6.0						7.3	5.4	2.5	4.1	4			
16	5.6	5	5.5						7.4	5.7	2.5	4.3	4.2		3.2	
17	5.6	6	5.6						7.6	6	2.6	4.5	4.3			
18	5.6	6	5.1						7.8	6.3	2.6	4.6	4.5			

Comparison of previously published lower reference limits for ferritin (µg/L) and the results of this study. Reference intervals for the parametric and polynomial analyses can be found in the supplementary table S1.

*Hb* hemoglobin, *MCV* mean corpuscular volume, *MCH* mean corpuscular hemoglobin, *MCHC* mean corpuscular hemoglobin concentration, *RDW *red cell distribution width.

**Table 3:** Lower reference limits (2.5th percentile) for ferritin in µg/L among pediatric patients for males.

Age	CLSI Parametric	CLSI Rank	Fractional polynomial	Correlation between ferritin and erythrocyte parameters					Hoq et al*.*^33^					Adeli et al*.*^11^		
				Hb	MCV	MCH	MCHC	RDW	Roche e602	Abbott	Roche c702	Siemens	Beckman	Abbott	Beckman	Ortho 5600
2	4.5	4	4.0	9	8	6	10	5	6.8	5.6	3.3	5.3	5.1	5	10.3	9.51
3	4.8	5	4.4						8.5	4.9	5.2	6.2	5.5			
4	6.4	6	6.4						9.9	4.9	6.8	6.9	5.9			
5	8.5	8	8.0	10	6	6	11	6	11	5.4	8	7.6	6.4	14		
6	9.2	9	9.1						12	6.1	9	8.1	6.8			
7	10.1	10	9.9						12.9	7	9.9	8.7	7.3			
8	11.8	11	10.5						13.7	8	10.7	9.2	7.8			
9	11.4	11	11.0						14.4	9.1	11.4	9.7	8.3			
10	13.4	14	11.4						15.1	10.3	12	10.1	8.8			
11	13.0	12	11.6						15.7	11.5	12.6	10.5	9.2			
12	12.2	12	11.9						16.3	12.7	13.1	10.9	9.7			
13	10.9	11	12.1	12	8	5	11	5	16.9	14	13.5	11.3	10.2			12.5
14	11.0	11	12.2						17.4	15.3	14	11.7	10.6			
15	12.2	11	12.3						17.9	16.6	14.4	12	11.1			
16	13.2	13	12.4						18.4	17.9	14.7	12.4	11.5		18.7	
17	15.1	14	12.5						18.8	19.3	15.1	12.7	12			
18	19.5	18	12.6						19.2	20.6	15.4	13.1	12.4			

Comparison of previously published lower reference limits for ferritin (µg/L) and the results of this study. Reference intervals for the parametric and polynomial analyses can be found in the supplementary table S1.

*Hb* hemoglobin, *MCV* mean corpuscular volume, *MCH* mean corpuscular hemoglobin, *MCHC* mean corpuscular hemoglobin concentration, *RDW* red cell distribution width.

**Figure 2:**
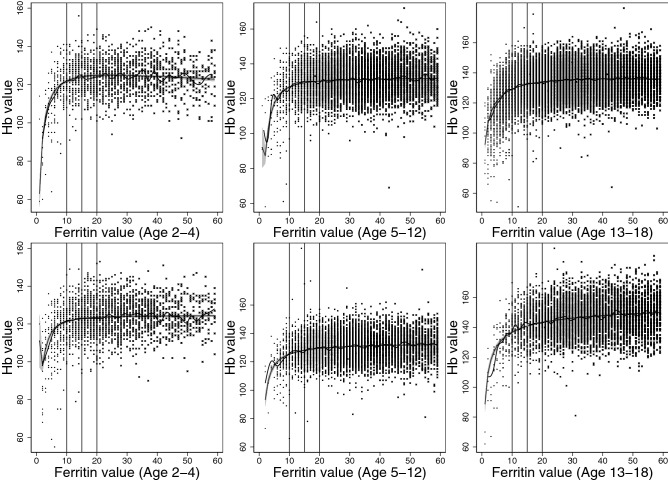
Median spline, scatterplot, and smoothed quantile regression based on 2-degree fractional polynomial models of the correlation between ferritin result values and hemoglobin (Hb) values. Results for females plotted on top, and results for males plotted on bottom for the three age groups, 2–4, 5–12, and 13–18 years.

**Figure 3:**
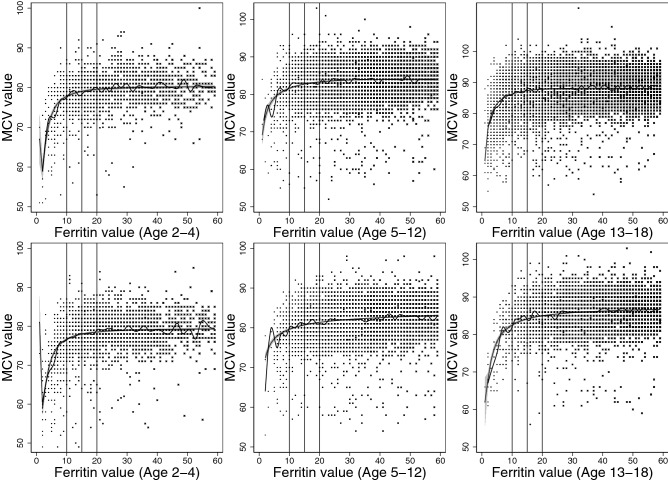
Median spline, scatterplot, and smoothed quantile regression based on 2-degree fractional polynomial models of the correlation between ferritin result values and mean corpuscular volume (MCV) values. Results for females plotted on top, and results for males plotted on bottom for the three age groups, 2–4, 5–12, and 13–18 years.

**Figure 4:**
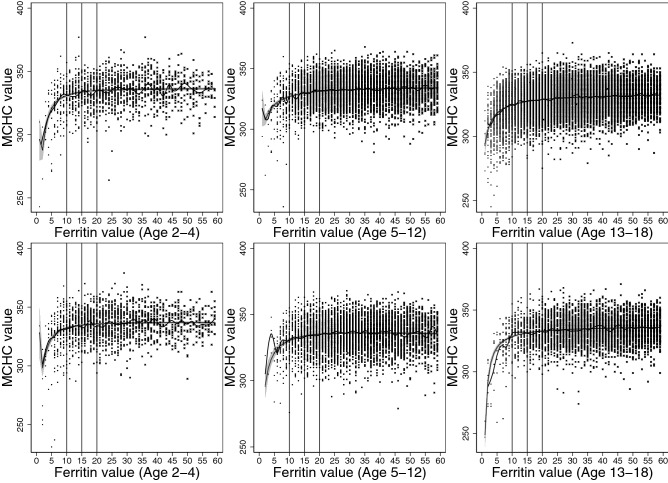
Median spline, scatterplot, and smoothed quantile regression based on 2-degree fractional polynomial models of the correlation between ferritin result values and mean corpuscular hemoglobin concentration (MCHC) values. Results for females plotted on top, and results for males plotted on bottom for the three age groups, 2–4, 5–12, and 13–18 years.

**Figure 5:**
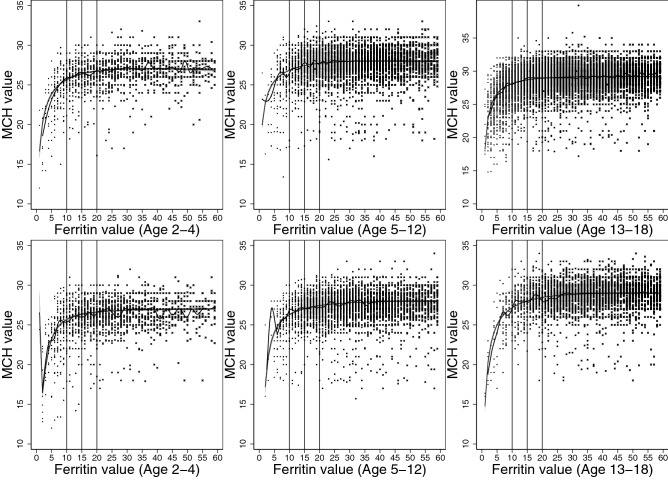
Median spline, scatterplot, and smoothed quantile regression based on 2-degree fractional polynomial models of the correlation between ferritin result values and mean corpuscular hemoglobin (MCH) values. Results for females plotted on top, and results for males plotted on bottom for the three age groups, 2–4, 5–12, and 13–18 years.

**Figure 6:**
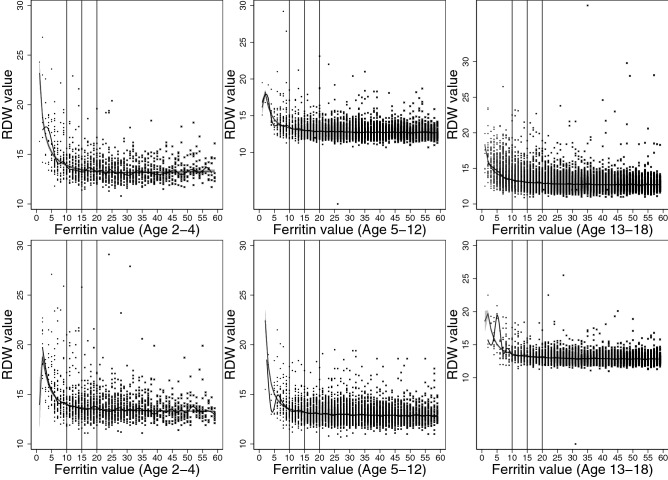
Median spline, scatterplot, and smoothed quantile regression based on 2-degree fractional polynomial models of the correlation between ferritin result values and red cell distribution width (RDW) values. Results for females plotted on top, and results for males plotted on bottom for the three age groups, 2–4, 5–12, and 13–18 years.

In our analysis of the correlation between ferritin and erythrocyte parameters, decrease in hemoglobin and MCHC levels occurred first, followed by MCV levels, and lastly, MCH and RDW levels. For hemoglobin, females presented with decreasing hemoglobin levels at 10 µg/L among younger children, and at 11 µg/L among adolescents (Table 2). For males, the level was slightly higher (12 µg/L) for adolescents, and slightly lower (9 µg/L) for young children (Table 3). MCHC levels started decreasing at a similar level to hemoglobin, near 10–11 µg/L for both genders. MCV levels started deteriorating at a lower corresponding value for ferritin, between 6 and 8 µg/L for both genders. Levels were lower for MCH and RDW: between 4 to 5 µg/L for females and 5–6 µg/L for males MCH, and 3–5 µg/L for females and 5–6 µg/L for males RDW. These results are graphically represented in Figs. 2, 3, 4, 5 and 6.

## Discussion

Generating pediatric reference intervals is a resource-intensive task. Laboratories frequently resort to using guideline-based diagnostic cut-offs or published reference intervals^29,30^. In our study, we used various approaches to derive a lower reference limit for ferritin from a large clinical laboratory dataset. Our results suggest that ‘functional’ reference limits based on disease progression (i.e. iron deficient erythropoiesis leading to anemia) may be more meaningful than commonly reported population distribution-based reference limits for nutritional biomarkers, such as ferritin.

In our analyses based on the conventional method of reporting population distribution based reference limits, we observed a decrease in the lower ferritin reference limit beginning with adolescence for females; which was also reported by Katayev et al*.*^31^ in their study using routinely collected data from laboratories, in the CALIPER study of healthy children^11^, and also in Australian studies by Southcott et al*.*^32^ and Hoq et al*.*^33^ on a healthy children (Table 2). In comparison, males had increasing lower ferritin reference limits by age (Table 3).

Females have a high prevalence of subclinical iron deficiency^34^, which could possibly be reflected into reference limits of these studies and ours. Southcott et al*.*^32^ compared their results to the CALIPER study, and suggested that a number of subjects in the CALIPER study could have had subclinical iron deficiency^32^. Although our study population was not representative of healthy children, our results were comparable to these studies. Therefore, the presence of subclinical iron deficiency could be affecting the reference intervals derived from conventional population distribution-based methods.

Our analysis on the correlation between ferritin and erythrocyte parameters, which was initially described by Markus et al*.*^18^*, *presents a functional lower reference limit that is associated with progression towards anemia. An advantage of this approach is that the reference limit is less influenced by potential subclinical iron deficiencies in the population. We observed a deterioration in hemoglobin concentration beginning near 10 µg/L ferritin, while MCV concentrations begin to decline at a lower ferritin level, of 7–8 µg/L. This is in line with the known progression of iron deficiency anemia: as iron deficiency remains, depleted iron stores results in a physiological response whereby a reduction of hemoglobin production occurs (i.e. lower hemoglobin MCH, and MCHC levels), which eventually leads to smaller erythrocytes (reduced MCV levels) and variable red cells widths (high RDW levels). Our results provide levels of serum ferritin correlating with this progression^35,36^.

Our results suggest that overall, a value of around 10 µg/L ferritin is the point at which iron deficiency anemia begins to occur. Anemia manifests late in clinical progression of iron deficiency erythropoiesis^6^. Therefore, the point of inflection for ferritin just prior to overt iron deficiency is expected to be higher than 10 µg/L^37^. Our results based on CLSI and fractional polynomial analyses, as well as other studies based on healthy children, suggested a reference limit lower than 10 µg/L. The definition and selection of healthy population in relation to iron metabolism is challenged by general lack of symptoms until the condition is relatively severe. This raises the question of whether the reference limit of nutritional markers should be based on functional limits or a fixed percentile of a reference (‘healthy’) population. Differences in results from functional correlation analysis of ferritin and hematological parameters, and population averaged analyses (i.e. CLSI and polynomial models), suggest the need for more nuanced consideration when deciding which methods are best suited to its clinical utility.

Using routinely collected data has limitations that should be considered. One limitation is the likely presence of unwell children, which has implications for population distribution based functional limits. We are unable to exclude unwell children with no record of their illness, or, in the case of inflammation, those without a CRP test. Previous studies have reported differences in reference values related to variation in laboratory methods^11,38^, and studies presenting indirect reference intervals are recommended to describe pre-analytical and analytical processes^13^. As our results are from general practices across a large region, the results would have been derived from a number of laboratories, possibly with different methods and analyzers. Determining and describing these procedures in our study is not possible as this information is not available. Nonetheless, our assessment of variation indicated that the results are stable across the study period, and does not present with significant variations. Although our results are similar to studies from other regions, it should be noted that our data comes from one state in Australia, and may not necessarily be representative of the general population. Larger studies in Australia, and replication of our methodology elsewhere would provide a greater understanding of ferritin levels in various regions. Our study has strengths, including the good overview from potentially healthy and ill patients, which is necessary for the correlation model described by Markus et al*.*^18^ to be fitted, and the large sample size from routinely collected data.

## Conclusion

Information-rich, routinely collected data sources provide an opportunity to correlate test results with patient outcomes and may be utilized in deriving or assessing clinical representativeness of reference intervals. Functionally derived reference limits with clinical utility provide a more meaningful reference limit for ferritin in diagnosing iron deficiency compared to commonly used population centiles. Ferritin concentration of 10 µg/L correspond to erythrocyte levels indicative of iron deficiency anemia. Therefore, the lower reference limit for ferritin should ideally be at a level above 10 µg/L, prior to progression to anemia.

## Supplementary information

Supplementary Information.

## Data Availability

De-identified individual participant data can be made available by the data custodians to researchers who provide a methodologically sound proposal for use in achieving the goals of their approved proposal, on condition of approval of the primary health networks on whose behalf the data is being collected for. Proposals can be submitted to Outcome Health at admin@outcomehealth.org.au.
