# Supplementary material for: Clinical thresholds for diagnosing iron deficiency: comparison of functional assessment of serum ferritin to population based centiles
Source: Sci Rep. 2020 Oct 26;10:18233. doi: 10.1038/s41598-020-75435-5 (PMC7589482; doi:10.1038/s41598-020-75435-5)
Supplement: Supplementary file 1 — Supplementary Information. [file 41598_2020_75435_MOESM1_ESM.pdf]

Clinical thresholds for diagnosing iron deficiency: comparison of functional assessment of serum ferritin to population based centiles

Gorkem Sezgin[1*], Paul Monagle[2,3,4], Tze Ping Loh[5], Vera Ignjatovic[2,3], Monsurul Hoq[2, 6], Christopher Pearce[7], Adam McLeod[7], Johanna Westbrook[1], Ling Li[1], Andrew Georgiou[1]

Affiliations: [1] Australian Institute of Health Innovation, Faculty of Medicine and Health Sciences, Macquarie University, Ryde, New South Wales, Australia, [2] Department of Pediatrics, The University of Melbourne, Parkville, Victoria, Australia, [3] Hematology Research, Murdoch Children's Research Institute, Parkville, Victoria, Australia, [4] Department of Hematology, Royal Children's Hospital Melbourne, Parkville, Victoria, Australia, [5] Department of Laboratory Medicine, National University Hospital, Kent Ridge, Singapore, [6] Clinical Epidemiology and Biostatistics Unit, Murdoch Children's Research Institute, Parkville, Victoria, Australia, [7.] Outcome Health, East Burwood, Victoria, Australia

Corresponding author contact: Gorkem Sezgin, Centre for Health Systems and Safety Research, Australian Institute of Health Innovation, Faculty of Medicine, Health and Human Sciences, Level 6, 75 Talavera Road, Macquarie University, NSW 2109, Australia, gorkem.sezgin@mq.edu.au , +61 2 9850 2940

Authors contact: Paul Monagle: paul.monagle@rch.org.au; Vera Ignjatovic: verai@unimelb.edu.au; Tze Ping Loh: tploh@hotmail.com; Monsurul Hoq: monsurul.hoq@mcri.edu.au; Christopher Pearce: drchrispearce@mac.com; Adam McLeod: AMcLeod@outcomehealth.org.au; Johanna Westbrook: johanna.westbrook@mq.edu.au; Ling Li: ling.li@mq.edu.au; Andrew Georgiou: andrew.georgiou@mq.edu.au

Supplemental table 1. Lower reference limits (2.5th percentile) for ferritin in µg/L among pediatric patients by gender, including their respective reference intervals.

Age	Female		Male	
	CLSI Parametric (90% CI)	Fractional polynomial (95% CI)	CLSI Parametric (90% CI)	Fractional polynomial (95% CI)
2	4.3 (3.9-4.8)	2.5 (1.9-3.1)	4.5 (4.1-4.9)	4 (2.6-5.4)
3	5 (4.5-5.4)	6.3 (5.9-6.6)	4.8 (4.5-5.2)	4.4 (3.6-5.2)
4	7.2 (6.8-7.7)	8 (7.7-8.3)	6.4 (6-6.8)	6.4 (5.8-7.1)
5	9.1 (8.6-9.7)	8.8 (8.6-9)	8.5 (8.1-8.9)	8 (7.5-8.5)
6	9.6 (9.1-10.1)	9.1 (8.9-9.3)	9.2 (8.8-9.7)	9.1 (8.7-9.5)
7	10.5 (10-11)	9.2 (9-9.4)	10.1 (9.6-10.6)	9.9 (9.6-10.3)
8	11.8 (11.3-12.4)	9 (8.8-9.2)	11.8 (11.3-12.2)	10.5 (10.2-10.8)
9	12.7 (12.1-13.3)	8.7 (8.5-8.9)	11.4 (10.8-12)	11 (10.7-11.3)
10	11 (10.5-11.6)	8.4 (8.2-8.5)	13.4 (12.9-14)	11.4 (11.1-11.6)
11	10.9 (10.5-11.4)	8 (7.8-8.1)	13 (12.5-13.6)	11.6 (11.3-11.9)
12	7.8 (7.4-8.2)	7.5 (7.4-7.6)	12.2 (11.7-12.8)	11.9 (11.6-12.2)
13	7.3 (7-7.7)	7 (6.9-7.1)	10.9 (10.5-11.4)	12.1 (11.7-12.4)
14	5.8 (5.6-6.1)	6.5 (6.4-6.6)	11 (10.5-11.4)	12.2 (11.9-12.5)
15	5.9 (5.7-6.1)	6 (5.9-6.1)	12.2 (11.6-12.7)	12.3 (12-12.7)
16	5.6 (5.4-5.8)	5.5 (5.4-5.6)	13.2 (12.6-13.8)	12.4 (12.1-12.8)
17	5.6 (5.4-5.7)	5 (4.9-5.1)	15.1 (14.4-15.9)	12.5 (12.2-12.9)
18	5.6 (5.4-5.8)	4.5 (4.3-4.6)	19.5 (18.7-20.4)	12.6 (12.3-13)

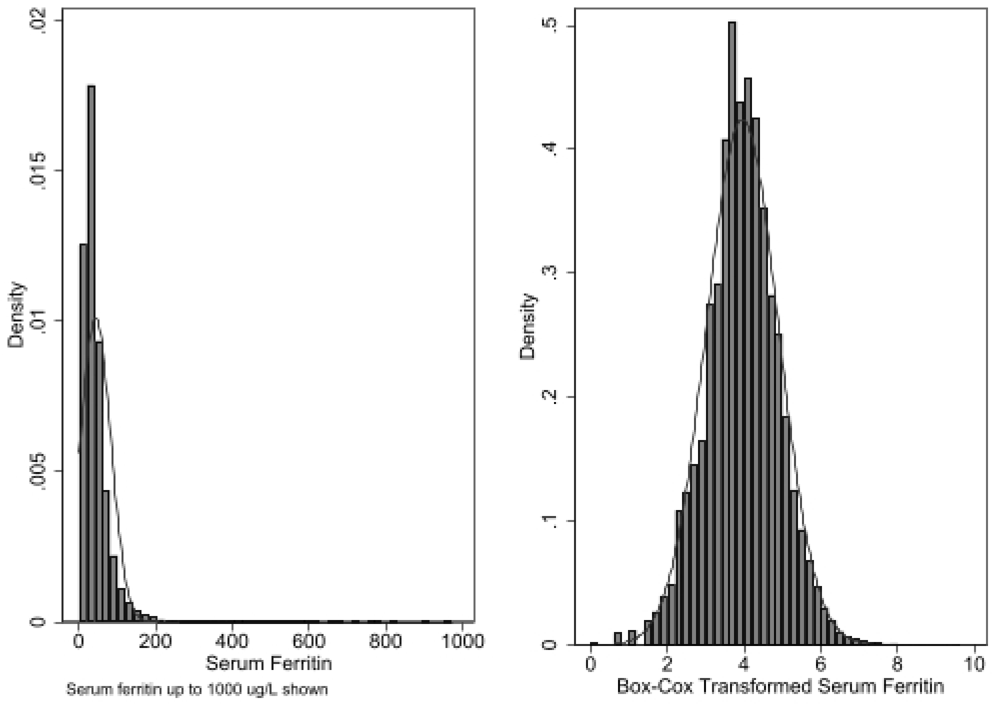

Supplemental Figure 1. Figure presenting the overall distribution of serum ferritin values, and an overall Box-Cox transformation reducing the skew of the distribution
